# Scanning electron microscopy datasets for local fibre volume fraction determination in non-crimp glass-fibre reinforced composites

**DOI:** 10.1016/j.dib.2021.106868

**Published:** 2021-02-12

**Authors:** Lars P. Mikkelsen, Søren Fæster, Stergios Goutianos, Bent F. Sørensen

**Affiliations:** Wind Energy Materials and Components, DTU Wind Energy, Technical University of Denmark, DK-4000 Roskilde, Denmark

**Keywords:** Bundle segmentation, SEM, Fatigue damage evolution, Wind turbine blades

## Abstract

The fatigue damage evolution depends on the local fibre volume fraction as observed in the co-submitted publication [1]. Conventionally, fibre volume fractions are determined as an averaged overall fibre volume fraction determined from small cuts of the laminate. Alternatively, automatically stitching of scanning electron microscopy (SEM) images can make high-resolution scans of large cross-section area with large contrast between the polymer and glass-fibre phase. Therefore, local distribution of the fibre volume fraction can be characterised automatically using such scan-data. The two datasets presented here cover two large Field of Views scanning electron microscopy (SEM) images. The two images is generated from between 1200 and 1800 high-resolution scan pictures which have been stitched into two high-resolution tif-files. The resolution corresponds to between 700 and 5000 pixels covering each fibre. The datasets are coming from two different non-crimp fabric glass fibre reinforced epoxy composites typically used in the wind turbine industry. Depending on the regions analysed, fibre volume fraction in the range of 50–85% is found. The maximum local fibre volume fraction is found averaging the local fibre volume fraction over 5 × 5 fibre diameter (80 × 80 µm^2^) areas. The local fibre volume fraction has been used in the analysis performed in [1].

## Specifications Table

**Table:** 

Subject	Material Science
Specific subject area	Composites, Scanning electron microscopy, fibre volume fraction
Type of data	Image (Scanning electron microscopy images) MetaData (hdr-files with metadata for the SEM data) Scripts (Matlab scripts for local fibre volume fraction determination)
How data were acquired	VEGA3 SBU tabletop microscope (SEM)
Data format	Raw: SEM individual micrographs in “.bmp” format zipped together Processed: SEM stitched micrographs in “.tif” format, Mat-files with the segmented areas. Analysed: Matlab scripts for analysing the SEM data
Parameters for data collection	The 2D micrographs were obtained using scanning electron microscope with an accelerating voltage of 20 kV.
Description of data collection	Cuts of two composites test samples embedded and prepared by standard grinding and polishing followed by SEM scanning.
Data source location	DTU Wind Energy, Roskilde, Denmark, Latitude: 55.695343, Longitude: 12.08921
Data accessibility	Repository name: Zenodo.org Data identification number: 4,064,835 Direct URL to data: http://doi.org/10.5281/zenodo.4064835
Related research article	B.F. Sørensen, S. Goutianos, L.P. Mikkelsen, S. Fæster, Fatigue damage growth and fatigue life of unidirectional composites. Composite Science and Technology, Submitted.

## Value of the Data

• The high-resolution large field of view SEM scanning data is used to determine the local fibre volume fraction distribution in two different non-crimp fabric based glass-fibre composites. The data is used to characterise local fibre volume fraction in conventional non-crimp fabrics, which values subsequently is used in reference [1].
• The industry and academia can use the provided datasets for studying fibre volume fraction variations in non-crimp fabric-based composites.
• The two datasets can be used as a benchmark dataset for developing segmentation and analysis tools for local fibre volume fraction and fibre diameter determination. The datasets can also be used for investigating variation in the fibre volume fractions at different locations in the fabric, e.g., close to the backing fibre bundle.

## 1 Data Description

For each of the two cases, five files are made available at the Zenodo repository, see [4]. Those two file-set contains:

• Tif-file: The stitched SEM scanned image which is used in the fibre volume fraction analysis
• Hdr-file: Meta-data about the stitched SEM scanned image
• M-file: The Matlab script used for analysing the tif-file
• Mat-file: Matlab mask data for a selection of the bundles used in the fibre volume fraction analysis
• Zip-file: Collection of the individual SEM scanned images and meta-data files behind the stitched SEM image.

**Fig. 1:**
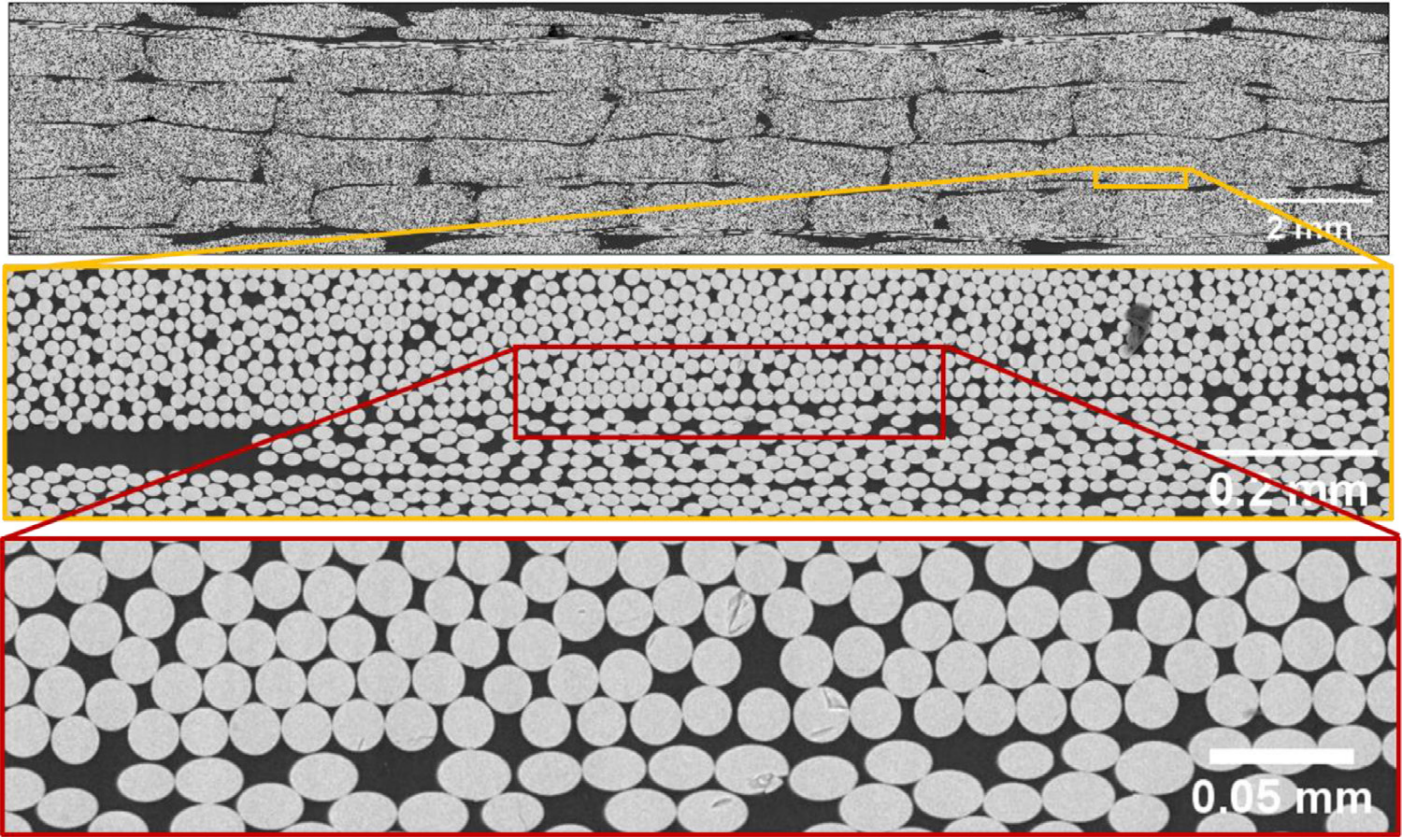
Scanning electron microscope images of Case 1 scanned with a VEGA3 SBU. With a pixel size of 527.25 nm, the fibre diameter (16 µm) will be cover 30 pixels over the diameter and 724 pixels over the fibre cross-section area.

**Fig. 2:**
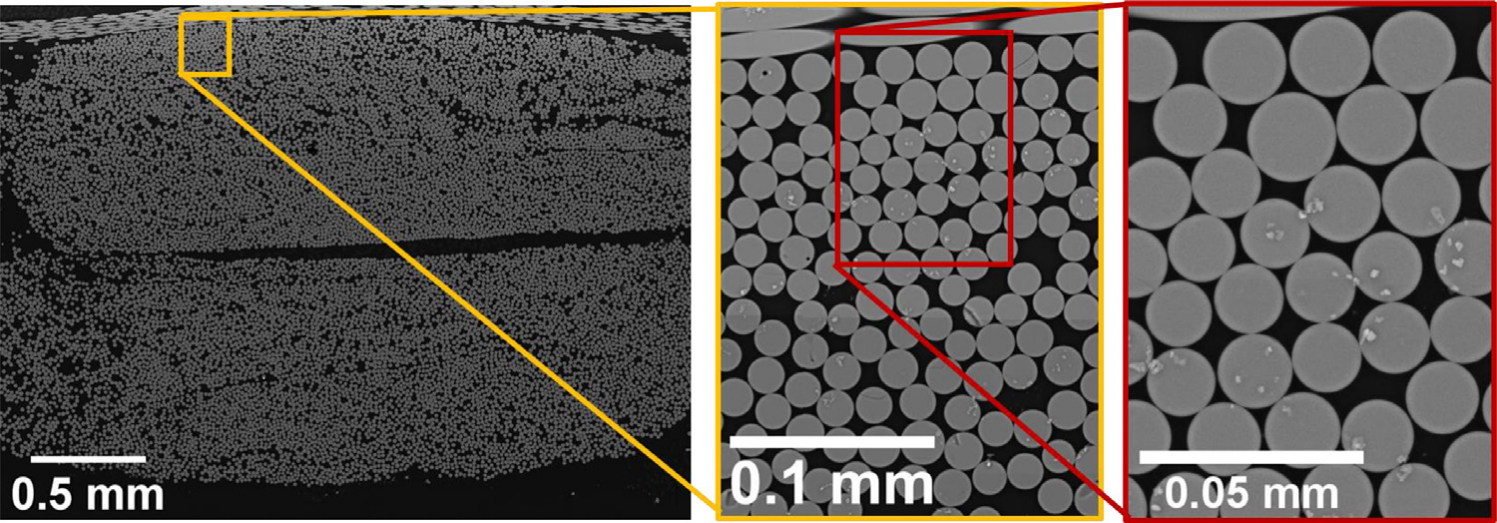
Scanning electron microscope images of case 1 scanned with a VEGA3 SBU. With a pixel size of 195.31 nm, the fibre diameter (16 µm) will be cover by 82 pixels over the diameter and 5300 pixels over the fibre cross-section area.

The full cross-section and a zoom-in on the scanning electron microscope images for the two cases are shown in Fig. 1, Fig. 2. The images were acquired using a Tescan VEGA3 SEM with the settings as listed in Table 1. The composite samples were cut orthogonal to the dominating fibre orientation, with the cutting surface, subsequently polished and applied with an approximately 10 nm thin layer of carbon using a Bal-Tec SCD 005 Sputter Coater. The material samples are cut from fatigue test samples used in reference [2] and [3]. The two cases will here be denoted as Cases 1 and 2, respectively. The images were taken with a pixel size of 527.25 nm and 195.31 nm for Figs. 1 and 2, respectively, using a source magnification of 692x at a high tension of 15 or 20 kV using a backscatter (BSE) detector.

**Table 1:** SEM data setting.

Parameter	Case 1	Case 2
Pixel size scan [nm]	390.63	195.31
Pixel size stitched [nm]	527.25	195.31
Magnification	692	692
Detector	BSE	BSE
Acc. voltage [kV]	15	20
Scan speed	6	6
Exposer time [µs/pxl]	32	32
Number of images	1764	1200
Overlap [%]	10	10
Total scan time [h]	15	6

## 2 Experimental Design, Materials and Methods

**Fig. 3:**
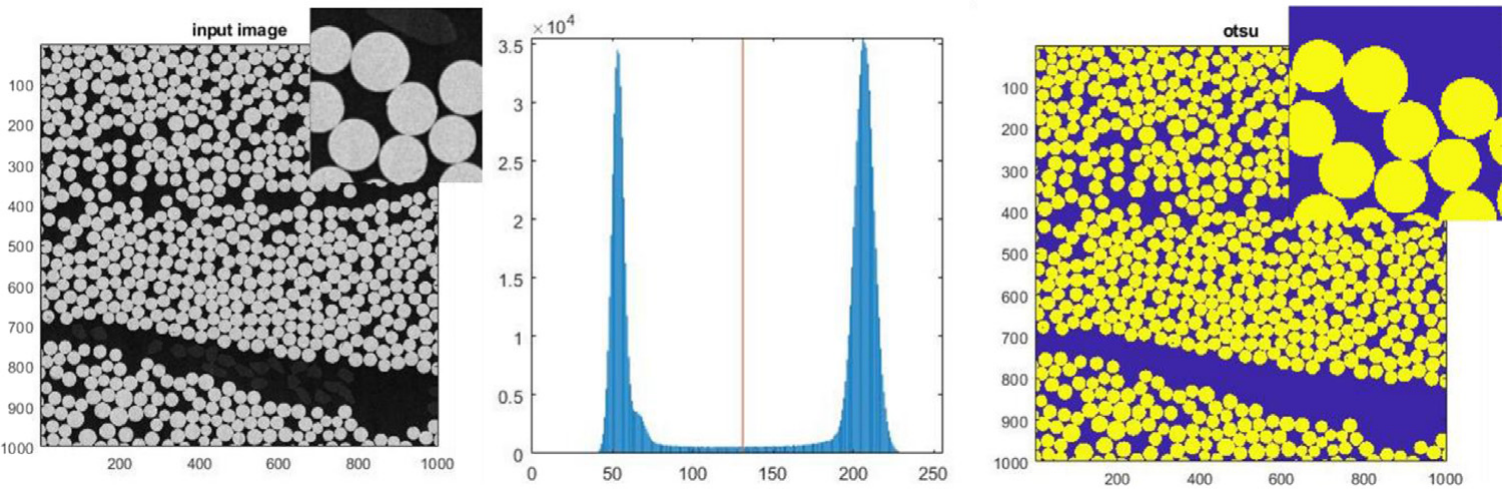
Otsu threshold for Case 1.

**Fig. 4:**
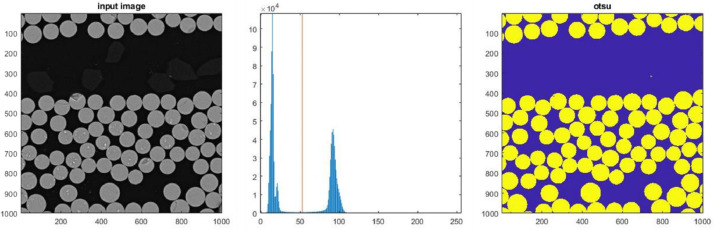
Otsu threshold for Case 2.

The scan parameter for the scanning electron microscopy (SEM) images, see Table 1, were carefully selected to generate the best possible contrast difference between fibres and resin. The SEM images were acquired and afterward stitched together with the “image snapper” function in the Tescan software VegaTC to generate SEM images of large regions with high resolution. The SEM images were processed with a MATLAB script, where the data were loaded with the function *imread* and binarised using an Otsu threshold value determined by the Matlab-function *otsuthresh* based on a histogram of a central 1000 × 1000 pixel subsection as shown in Figs. 3 and 4 for Cases 1 and 2, respectively.

The binarisation shown to the left in Figs. 3 and 4 can now be used for calculating the fibre volume fraction simply by finding the ratio between the number of binarised pixels above the Otsu threshold (fibers) over the total amount of pixels inside the region. For the total scanned area shown in Figs. 1 and 2, the overall fibre volume fraction is found to be $V_{f}=0.598$ and $V_{f}=0.525$, respectively. A value that in Table 2 is compared with the values measured by a back-calculated or burn-of experiment reported in the two references [2] and [3], respectively. For Case 1, it should be noted that not the total layer of the lower biax ply is included in the SEM scan, which may result in the slightly larger overall fibre volume fraction found compared to the value reported in reference [2].

**Table 2:** Fibre volume fractions.

		Overall $V_{f}$			
Case	Reference	From ref.	SEM	Bundle $V_{f}$	Max $V_{f}$$5D_{f}\times \;5D_{f}$
Case 1	[2]	0.57	0.598	0.668 ± 0.020	≈0.85
Case 2	[3]	0.53	0.525	0.606 ± 0.042	≈0.85

**Fig. 5:**
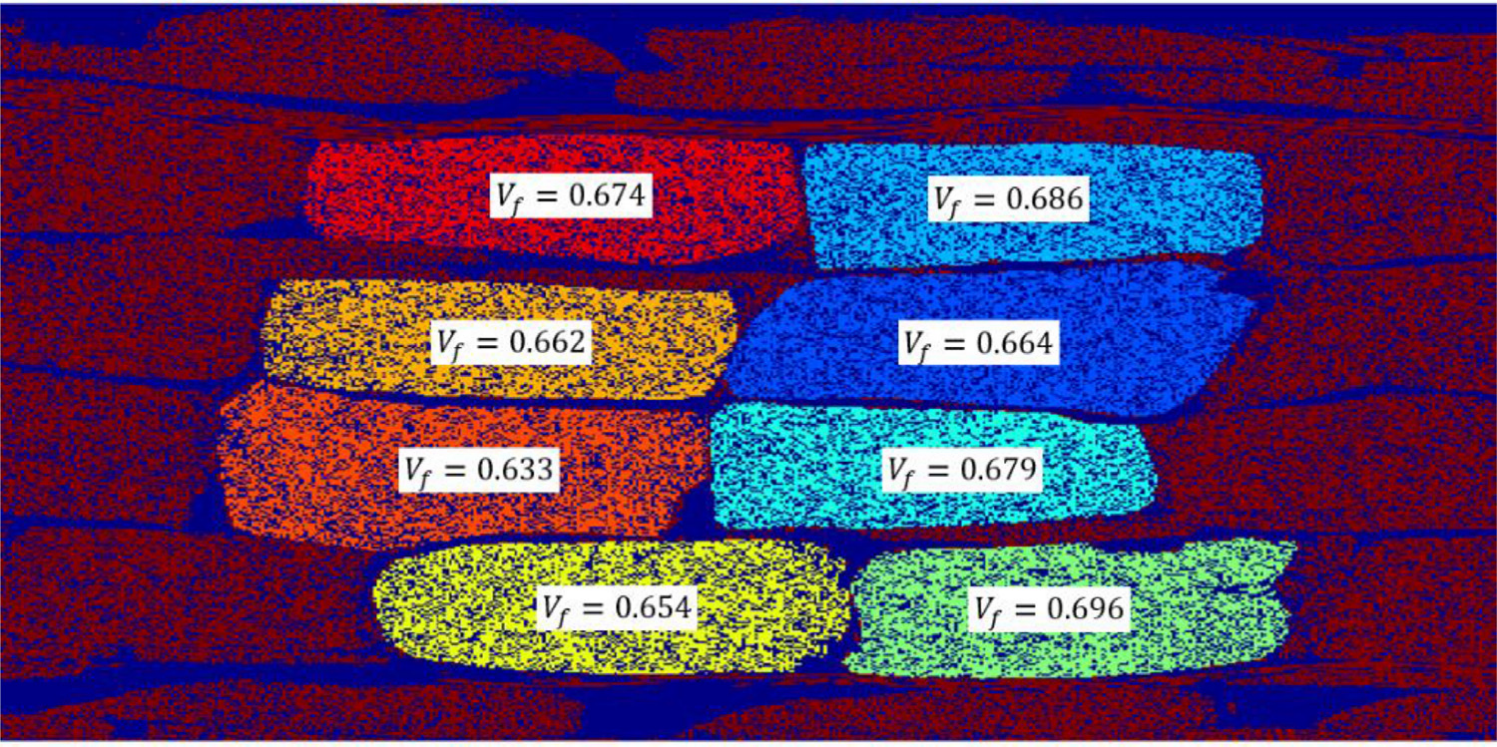
Local fibre volume fraction of 8 UD bundles from a zoomed region of Case 1.

**Fig. 6:**
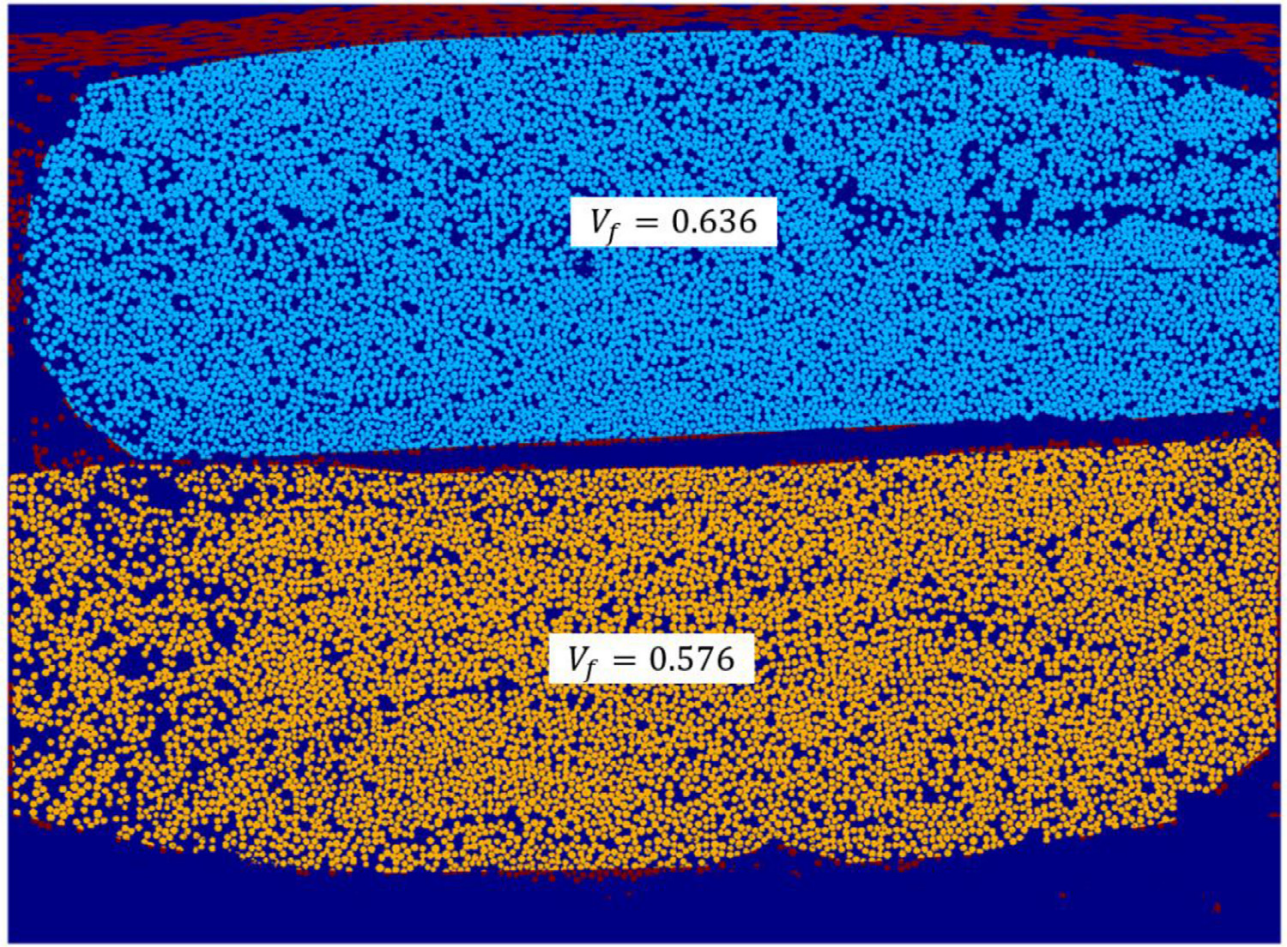
Local fibre volume fraction of 2 UD bundles from Case 2.

Fig. 5, Fig. 6 show manually segmented UD fibre bundles from inside the SEM scanned region. In Case 1, 8 different unidirectional bundles are segmented, while it for Case 2 includes a major part of two unidirectional bundles. The unidirectional bundles were manually segmented using the Region of Interest drawing tool in the Image Segmenter toolbox in Matlab. Inside each segmented region, the fibre volume fraction was calculated in the same way as for the overall fibre volume fraction. The values are reported in Table 2 together with their standard deviations.

**Fig. 7:**
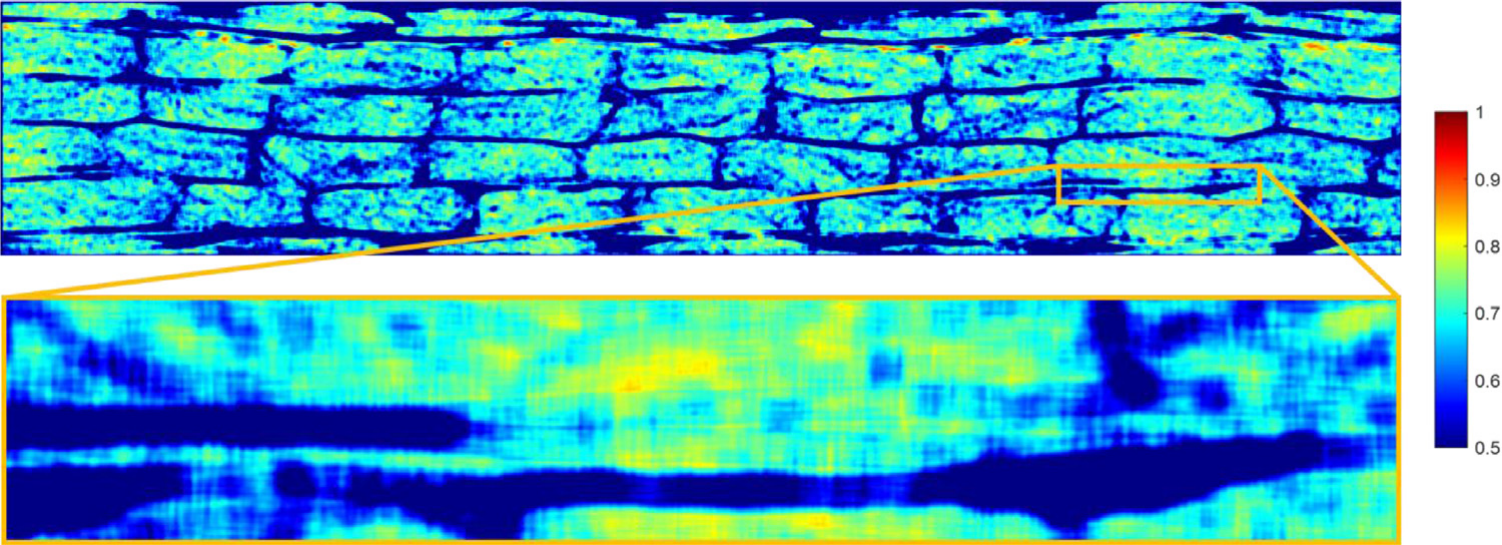
Averaging over a 5 × 5 fibre diameters moving area for Case 1.

**Fig. 8:**
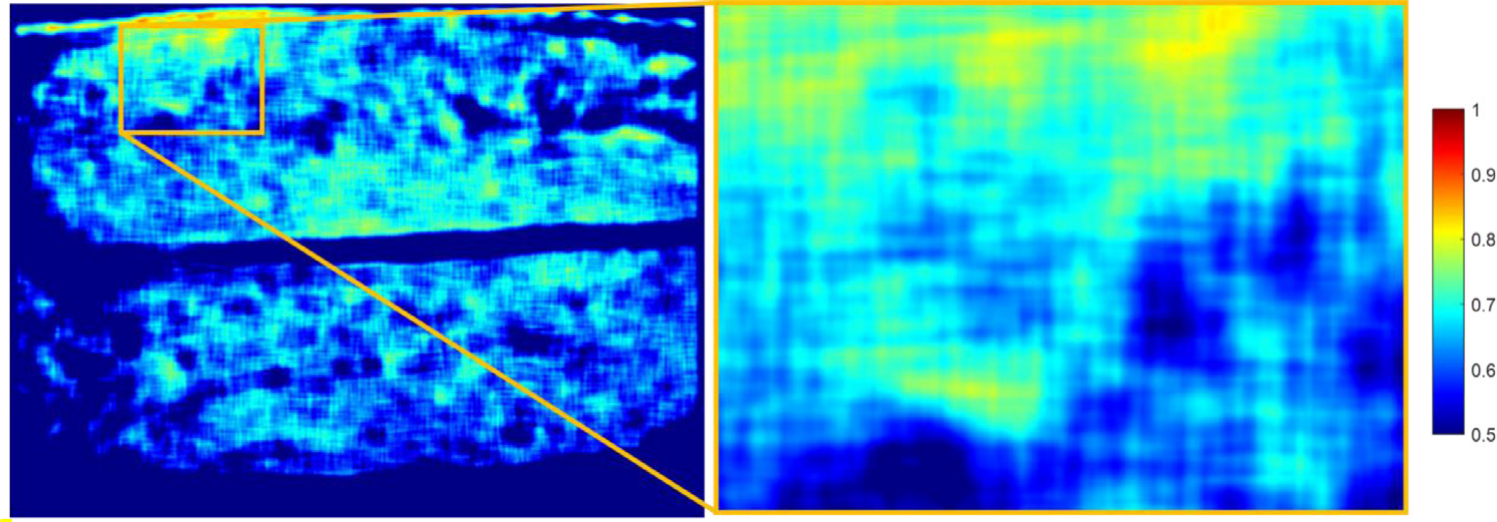
Averaging over a 5 × 5 fibre diameters moving area for Case 2.

Figs. 7 and 8 show the variation of the fibre volume fraction calculated by using a moving area averaging of a 5 × 5 fibre diameters sized area which for a fibre diameter equal to $D_{f}=16\mu m$ corresponds to approximately 80μm×80μm . This was done using the Matlab function *conv2* and was applied over the full scanned region as shown in Figs. 7 and 8. From this, a local fibre volume fraction is determined. In addition to the full scanned region, an image of a region with regions of high fibre volume fraction is presented. From the contour plots, regions with local fibre volume fraction up to around $V_{f}\approx 0.85$ for both cases were identified.

## CRediT Author Statement

**Lars P. Mikkelsen**: Conceptualization, Software, Visualization, Writing – Original Draft, Writing – Review & Editing; **Søren Fæster**: Methodology, Visualization, Writing – Review & Editing; **Stergios Goutianos**: Writing – Review & Editing; **Bent F. Sørensen**: Conceptualization, Funding acquisition, Writing – review & editing.

## Declaration of Competing Interest

The authors declare that they have no known competing financial interests or personal relationships which have or could be perceived to have influenced the work reported in this article.
